# Zi Shen Huo Luo Formula Prevents Aldosterone-Induced Cardiomyocyte Hypertrophy and Cardiac Fibroblast Proliferation by Regulating the Striatin-Mediated MR/EGFR/ERK Signaling Pathway

**DOI:** 10.1155/2020/9028047

**Published:** 2020-09-16

**Authors:** Weike Feng, Yue Zhao, Xiaotong Song, Yuan Wang, Qian Chen, Haijun Zhao, Guangying Lu, Hongyan Yuan, Zhichun Wu, Huayun Yu

**Affiliations:** ^1^Shandong University of Traditional Chinese Medicine, Jinan 250355, China; ^2^Shandong Co-Innovation Center of Classic TCM Formula, Jinan 250355, China; ^3^Shandong Institute of Commerce & Technology, Jinan 250103, China

## Abstract

Inappropriate activation of the renin-angiotensin-aldosterone system (RAAS) is an important factor in the development of hypertension. Excessive aldosterone can lead to myocardial extracellular matrix collagen proliferation, fibrosis, and cardiomyocyte hypertrophy and aggravate maladaptive remodeling. The results of our previous clinical and animal experiments suggested that Zi Shen Huo Luo Formula (ZSHLF) combined with perindopril can effectively control the process of left ventricular hypertrophy (LVH). The purpose of this study was to investigate whether ZSHLF-treated serum inhibits the membrane localization of the striatin-mediated mineralocorticoid receptor (MR) and affects MR-mediated nongenomic effects and the downstream epidermal growth factor receptor (EGFR)/extracellular regulated kinase (ERK) signaling pathways, thereby improving aldosterone-induced myocardial remodeling. Serum containing ZSHLF was prepared and used to treat rat cardiomyocytes and cardiac fibroblasts in vitro after aldosterone induction and striatin knockdown by small interfering RNA (siRNA). Cell-based assays were carried out to determine the cardiomyocyte surface area and assess the proliferation rate and hydroxyproline secretion of cardiac fibroblasts. Quantitative real-time PCR (qRT-PCR), immunoprecipitation (IP), and Western blotting were performed to evaluate the striatin-mediated MR/EGFR/ERK signaling pathway. In the present study, ZSHLF attenuated the aldosterone-induced hypertrophy of cardiomyocytes and inhibited the proliferation and collagen synthesis of cardiac fibroblasts. ZSHLF also reduced striatin mRNA expression and inhibited striatin and MR binding, membrane MR protein expression, and EGFR and ERK1/2 phosphorylation. Furthermore, after striatin silencing with siRNA, some of the effects of ZSHLF were not changed significantly. In conclusion, ZSHLF inhibits the downstream EGFR/ERK signaling pathway by blocking the striatin-mediated membrane localization of MR, which may be an important molecular mechanism by which ZSHLF improves aldosterone-induced myocardial remodeling.

## 1. Introduction

Hypertension is one of the most common noncommunicable diseases in the world, and approximately 1 billion patients have currently been diagnosed with hypertension worldwide. Left ventricular hypertrophy (LVH), the most common complication of hypertension, is mainly characterized by cardiomyocyte hypertrophy and the disproportionate proliferation of collagen fibers, and it evolves into progressive left ventricular dysfunction and heart failure [1, 2]. The renin-angiotensin-aldosterone system (RAAS), a hormonal signaling cascade with broad activity in the heart, blood vessels, kidney, and adrenal gland, plays a critical role in maintaining normal blood pressure and electrolyte balance [3, 4]. Angiotensin converting enzyme inhibitors (ACEIs) are some of the most commonly used drugs in the treatment of hypertensive LVH. However, excessive aldosterone levels caused by long-term treatment with ACEIs can cause myocardial extracellular matrix deposition, fibrosis, and cardiomyocyte hypertrophy and affect myocardial remodeling [5, 6].

Recent studies have shown that the rapid nongenomic effects of aldosterone are closely related to aldosterone-induced myocardial remodeling [7, 8]. Aldosterone binds and activates the cytoplasmic mineralocorticoid receptor (MR), which acts as a nuclear transcription factor to induce the expression of specific mRNAs [9, 10]. However, the nongenomic effects of MR have been confirmed to originate from MR near the plasma membrane, although MR may not be directly inserted into the plasma membrane because MR lacks palmitoylation sites similar to those identified as transmembrane domains in other steroid receptors [11, 12]. Striatin is an important mediator of the nongenomic effects of aldosterone. Aldosterone can bind MR attached to the plasma membrane through scaffold proteins, such as striatin and Cav1, and interacts with receptor tyrosine kinases, such as the epidermal growth factor receptor (EGFR), platelet-derived growth factor receptor, insulin-like growth factor 1 receptor, angiotensin II receptor 1, and G1 protein-coupled receptors, to mediate nongenomic effects through MR [13, 14].

Zi Shen Huo Luo Formula (ZSHLF) is an empirical prescription for the treatment of hypertensive LVH that contains 6 traditional Chinese medicine compounds. Previous studies suggested that combined treatment with ZSHLF and the ACEI perindopril reduced the left ventricular mass index, reversed left ventricular remodeling [15], corrected the imbalance between type I collagen synthesis and degradation, inhibited cardiac hypertrophy and cardiac fibrosis in spontaneously hypertensive rats (SHRs), and effectively regulated the process of LVH. However, the molecular mechanism of ZSHLF in the treatment of hypertensive LVH is still unclear. In this study, we observed the nongenomic effects of aldosterone on cardiomyocyte hypertrophy, cardiac fibroblast proliferation, and collagen synthesis. We furthermore observed the effects of ZSHLF on aldosterone-induced myocardial remodeling, the interaction between striatin and MR, and the expression of downstream EGFR/extracellular regulated kinase (ERK) signaling pathway molecules. The purpose of this study was to investigate whether ZSHLF affects the nongenomic effects of aldosterone and improves myocardial remodeling by regulating the striatin-mediated MR/EGFR/ERK signaling pathway.

## 2. Materials and Methods

### 2.1. Preparation of ZSHLF-Containing Serum

All studies were carried out following the guidelines of the National Research Council of China and approved by the Animal Ethics Committee of Shandong University of Traditional Chinese Medicine. The herbal components of ZSHLF were purchased from the Shandong Hospital of Traditional Chinese Medicine (Jinan, China). The following 6 crude drug materials fixed at a ratio of 20 : 15 : 12 : 12 : 20 : 3 were boiled twice in an 8-fold volume of ddH_2_O for 1 h per wash: *Scrophularia ningpoensis* Hemsl., *Achyranthes bidentata* Bl., the dried rhizome of *Coptis chinensis* Franch., the dried root cortex of *Paeonia suffruticosa* Andr., the dried aerial part of *Leonurus japonicu*s Houtt., and the dried bark of *Cinnamomum cassia* Presl., respectively. Twenty Sprague-Dawley (SD) rats (half male and half female, weighing 200 ± 20 g) were purchased from the Beijing Weitong Lihua Experimental Animal Center (Beijing, China; license No. SCXK (Jing) 2012-0001). The rats were randomly divided into two groups of 10 rats each, with one group receiving ZSHLF at a dose of 8.2 g/kg by intragastric administration twice a day for 5 days and the other group receiving the same amount of normal saline. The rats were starved for 12 h before the last administration of ZSHLF or saline and then given the dose for one day. One hour after the last drug administration, blood was collected, and then, the serum was separated, inactivated at 56°C, and filtered with a 0.22 *μ*m membrane. The serum was kept frozen at −80°C until use.

### 2.2. Identification of the Main Chemical Constituents of ZSHLF-Containing Serum

The main chemical components were identified using an UltiMate 3000 RS ultraperformance liquid chromatography (UPLC) system coupled to a Q-Exactive mass spectrometer (Thermo Fisher Scientific, MA, USA). Each serum sample (200 *μ*l) was mixed well with 1 ml of methanol: water (8 : 2, v : v), vortexed, and centrifuged for 10 min at 20000 ×g and 4°C. The supernatant was filtered through a 0.22 *μ*m membrane, and the filtrate was analyzed with a computer. The analytes were separated on a Welch XB-C18 UPLC column (250 mm × 4.6 mm, 5 um) at 35°C under the following conditions: aqueous phase, 0.1% formic acid aqueous solution; organic phase, 0.1% formic acid in acetonitrile; and flow rate, 1.2 ml/min. The mass spectrometer parameters were set as follows: ion source, electrospray ionization (ESI); scanning mode, positive and negative ion switching; resolution, 70000 (full mass) and 17500 (dd-MS2); scanning range, 100.0–1500.0 m/z; spray voltage, 3.8 kV (positive); capillary temperature, 300°C; collision gas, high purity argon; sheath gas, nitrogen, 40 Arb; auxiliary gas (auxiliary gas heater temperature), nitrogen, 350°C; and data acquisition time, 30.0 min.

### 2.3. Cell Culture and Treatment

H9c2 (2-1) rat cardiomyocytes were purchased from the Shanghai Cell Bank of the Chinese Academy of Sciences (Shanghai, China); rat cardiac fibroblasts were purchased from Wuhan Punuosai Life Technology (Wuhan, China). Cardiomyocytes and cardiac fibroblasts were cultured in Dulbecco's modified Eagle's medium (DMEM) (Invitrogen, CA, USA) containing 10% fetal bovine serum (GIBCO, MD, USA), 1% penicillin, and streptomycin mixture in a 5% CO_2_ incubator at 37°C. To observe the effect of aldosterone, we incubated cardiomyocytes with 10^−9^ mol/l aldosterone and cardiac fibroblasts with 10^−7^ mol/l aldosterone. To observe the effect of ZSHLF, we pretreated the two cell types with ZSHLF-containing serum for 2 h and then incubated the cells with aldosterone at the above concentrations.

### 2.4. Observation and Measurement of the Cardiomyocyte Surface Area

Cardiomyocytes were collected and treated with 4% paraformaldehyde, 0.5% Triton X-100, and 3% methanol hydrogen peroxide at room temperature for 20 min, and then, they were treated with a sealing solution containing goat serum at room temperature for 20 min. The cells were incubated with anti-*α*-actin primary antibody (1 : 60, Abcam, UK) overnight at 4°C and then incubated with fluorescein isothiocyanate- (FITC-) labeled secondary antibody (Beijing Solaibao Technology, China) for 20 min at 37°C. Finally, the cells were stained with 4′,6-diamidino-2-phenylindole (DAPI) (1 : 1000, Beijing Solaibao Technology, China) for 10 min and observed and photographed with an IX71 fluorescence microscope (Olympus Corporation, Japan). The surface area of all cells in the visual field was measured by ImageJ software, and the average values were taken as the results.

### 2.5. Detection of the Proliferation of Cardiac Fibroblasts

Cell Counting Kit-8 (CCK-8) reagent (SAB, MD, USA) and serum-free basic medium were mixed at a 1 : 10 volume ratio and added to the cardiac fibroblasts, which were incubated in a 5% CO_2_ incubator at 37°C for 1 h. The absorbance at a wavelength of 450 nm was determined with an MK3 microplate reader (Thermo Fisher Scientific).

### 2.6. Detection of Collagen Synthesis in Cardiac Fibroblasts

The hydroxyproline content in the cell culture medium was used to evaluate collagen production in the cardiac fibroblasts [16], and it was measured according to the instructions of a hydroxyproline kit (Nanjing Construction Bioengineering Research Institute, China). A UV-5200 ultraviolet spectrophotometer (Shanghai Yuanxi Instrument, China) was used to determine the absorbance at a wavelength of 550 nm. The hydroxyproline content was calculated and used to detect collagen synthesis.

### 2.7. Immunoprecipitation (IP)

Cardiomyocytes and cardiac fibroblasts were lysed with NET cell buffer (containing 1 mM PMSF and 1 mM protease inhibitor) by ultrasound on ice for 5 min. The lysate was centrifuged at 12000 ×g for 20 min at 4°C. The supernatant combined with lysis buffer (20 mM Tris-HCl, pH 7.5, 150 mM NaCl, 1% Triton X-100, 1 mM EDTA, and protease inhibitor) was centrifuged at 13000 rpm for 10 min at 4°C. The protein content of the supernatant was quantified by the bicinchoninic acid (BCA) method (4–8 *μ*g/*μ*l). Proteins (500 *μ*g) were incubated with primary antibody (antistriatin, anti-MR, 1 : 60, Abcam, UK) for precipitation overnight at 4°C and then incubated with protein G-agarose beads overnight at 4°C. The mixture was centrifuged at 2500 rpm for 1 min at 4°C, and the sediment was supplemented with protein sample buffer, boiled for 5 min, and centrifuged at 2500 rpm for 1 min. Proteins in the supernatant were detected by Western blotting.

### 2.8. Small Interfering RNA (siRNA) Transfection

Three siRNAs against striatin (siRNA STRs) were synthesized by Shanghai Jima Pharmaceutical Technology (Shanghai, China). The si-striatin-1 target sequence was GCAGACTCATTAGCTTATG, the si-striatin-2 target sequence was GCGCTGACTGTATTTAATG, and the si-striatin-3 target sequence was GCAGCGAATTCTCACGTTA. Nontarget siRNA was used as a negative control (NC) sequence. Cardiomyocytes and cardiac fibroblasts were transfected with the three siRNAs against striatin according to a previously described method [17], and cells were collected for PCR to identify the interference efficiency (Supplementary Figure 1).

### 2.9. Quantitative Real-Time PCR (qRT-PCR)

Primer sequences were designed based on GenBank sequences with Primer Premier 5.0 software (Table 1). The PCR primers were synthesized by Shanghai Jima Pharmaceutical Technology (Shanghai, China). mRNA was extracted using TRIzol (Thermo Scientific), and RNA was reverse transcribed with a TIANScript RT Kit (Tiangen Biotechnology, Beijing, China) according to the manufacturer's instructions. The reaction system was prepared with a SYBR Green PCR kit (Tiangen Biotechnology), and mRNA amplification and quantification were performed using an ABI 7500 fluorescence quantitative PCR instrument (Applied Biosystems, Foster, USA) according to the instructions. The reaction procedure was as follows: preincubation at 95°C for 5 min, amplification at 95°C for 10 s and 60°C for 60 s for 40 cycles, and separation at 95°C for 10 s and 60°C for 60 s. Target gene expression was normalized to the expression of glyceraldehyde-3-phosphate dehydrogenase (GAPDH), and relative expression was determined using the 2^−ΔΔct^ method.

### 2.10. Western Blotting

Cardiomyocytes and cardiac fibroblasts were collected and lysed to extract the proteins. The membrane proteins were extracted with a cell membrane protein extraction kit (Biyuntian Institute of Biotechnology, Shanghai, China) following the manufacturer's instructions. The protein concentration was detected using a BCA assay. The proteins were resolved by 10% sodium dodecyl sulfate polyacrylamide gel electrophoresis (SDS-PAGE) and transferred to polyvinylidene fluoride (PVDF) membranes. Then, the PVDF membranes were blocked with 5% skim milk for 1 h and incubated with antibodies against MR (1 : 60, Abcam), EGFR (1 : 1200, Abcam), pEGFR (1 : 800, Abcam), ERK1/2 (1 : 1000, Abcam), pERK1/2 (1 : 600, Cell Signaling Technology, Inc., USA), Na-K ATPase (1 : 1000, Abcam), and *β*-actin (1 : 1000, Abcam) overnight at 4°C. After the membranes were washed with TBST, they were incubated with secondary antibodies for 4 h at room temperature. Finally, immunoreactivity was visualized with an ECL kit (Millipore, MA, USA). The relative expression levels (ratio compared to Na-K ATPase or *β*-actin expression) of the target proteins were quantified by ImageJ software.

### 2.11. Statistical Analysis

The results are presented as the mean ± standard deviation of at least three independent experiments. Data were analyzed by one-way ANOVA for multiple comparisons with SPSS 22.0 software. Differences were considered statistically significant at *P* < 0.05.

## 3. Results

### 3.1. Analysis of the Main Chemical Components of ZSHLF-Containing Serum

**Figure 1:**
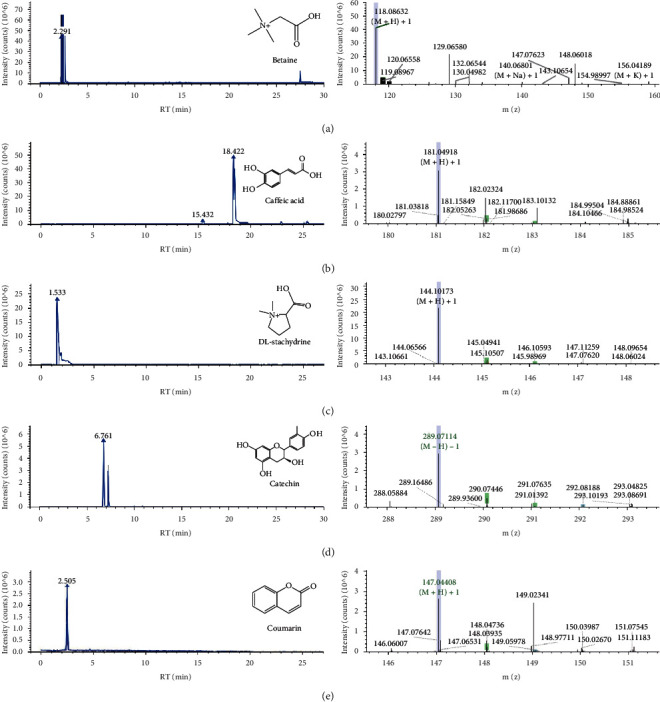
UPLC chromatograms and mass spectrometry results for ZSHLF-containing serum. Betaine (a), caffeic acid (b), DL-stachydrine (c), catechin (d), and coumarin (e) were identified as the main chemical components of ZSHLF.

As shown in Figure 1, UPLC was used to analyze the ZSHLF-containing serum, and mass spectrometry was used to identify the five main chemical components: betaine, caffeic acid, DL-stachydrine, catechin, and coumarin.

### 3.2. Effects of Aldosterone Induction Time on Cardiomyocyte Surface Area and the Proliferation and Hydroxyproline Secretion of Cardiac Fibroblasts

**Figure 2:**
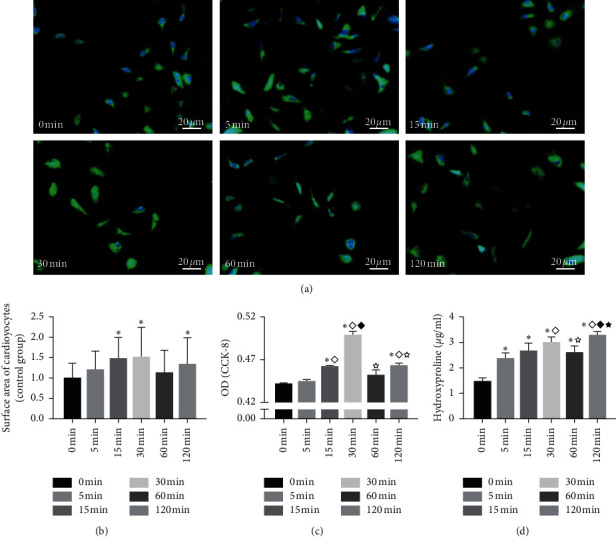
Effects of aldosterone induction time on the cardiomyocyte surface area and proliferation and hydroxyproline secretion of cardiac fibroblasts. Cardiomyocytes were treated with 10^−9^ mol/l aldosterone, and cardiac fibroblasts were treated with 10^−7^ mol/l aldosterone for 0, 5, 15, 30, 60 or 120 min. (a) Cells were immunofluorescence stained with DAPI (blue), and cardiomyocytes were detected by immunofluorescence staining using FITC-conjugated *α*-actinin (green). (b) Cardiomyocyte surface area (relative to that in the untreated group) was determined by *α*-actinin immunofluorescence staining. (c) The OD values of cardiac fibroblasts were detected by a CCK-8 assay. (d) Hydroxyproline content in the cardiac fibroblast culture supernatant. ^*∗*^*P* < 0.05 vs. the untreated group; ^◇^*P* < 0.05 vs. the group treated for 5 min; ^◆^*P* < 0.05 vs. the group treated for 15 min; ^☆^*P* < 0.05 vs. the group treated for 30 min; and ^★^*P* < 0.05 vs. the group treated for 1 h.

As shown in Figure 2(a), *α*-actinin immunofluorescence staining revealed adherent cardiomyocytes with a variety of shapes, including fusiform and polygonal cardiomyocytes. The cardiomyocyte surface area after induction with aldosterone for 15 min, 30 min, and 2 h was significantly greater than that of uninduced cardiomyocytes (*P* < 0.05), with the most obvious increase observed in the group treated for 30 min (Figure 2(b)).

In the CCK-8 assay, which was used to detect the proliferation of cardiac fibroblasts, the optical density (OD) values of the groups induced with aldosterone for 15 min, 30 min, and 2 h were significantly higher than that of the uninduced group (*P* < 0.05). The OD value of the group induced with aldosterone for 30 min was significantly higher than that of the other groups (*P* < 0.05) (Figure 2(c)).

Detection of the hydroxyproline content in the cardiac fibroblast culture supernatant revealed a significant increase in hydroxyproline secretion after 5 min of aldosterone induction (*P* < 0.05), and the hydroxyproline concentration of each group induced with aldosterone was significantly higher than that of the uninduced group (*P* < 0.05). The hydroxyproline content was significantly higher after 30 min of induction than after 15 min and 1 h of induction and after 2 h of induction than after 5 min, 15 min, and 1 h of induction (*P* < 0.05) (Figure 2(d)).

These results suggested that 30 min of aldosterone treatment induced substantial cardiomyocyte hypertrophy and increased the proliferation and hydroxyproline secretion of cardiac fibroblasts. These short-term effects are consistent with nongenomic effects. Because this study aimed to explore the nongenomic effects of aldosterone, an aldosterone induction time of 30 min was used in the following experiments.

### 3.3. Effects of ZSHLF on the Cardiomyocyte Surface Area and the Proliferation and Hydroxyproline Secretion of Cardiac Fibroblasts

**Figure 3:**
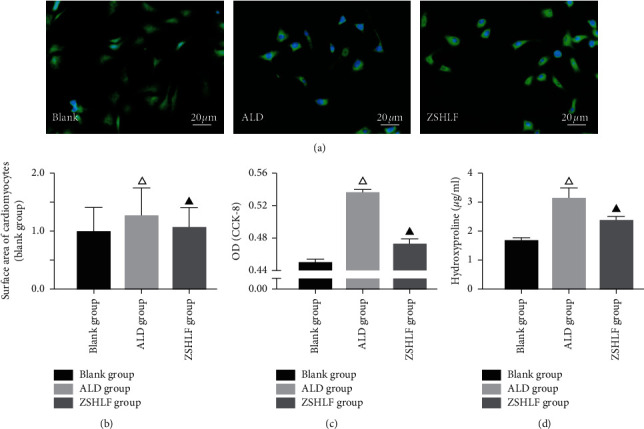
Effects of ZSHLF on the cardiomyocyte surface area and the proliferation and hydroxyproline secretion of cardiac fibroblasts. Cardiomyocytes and cardiac fibroblasts in the control group were pretreated with blank serum. In the aldosterone (ALD) group, cardiomyocytes and cardiac fibroblasts were pretreated with blank serum, and the cardiomyocytes were then incubated with 10^−9^ mol/l aldosterone for 30 min, and the cardiac fibroblasts were incubated with 10^−7^ mol/l aldosterone for 30 min. In the ZSHLF group, cardiomyocytes and cardiac fibroblasts were pretreated with 10% ZSHLF-containing serum for 2 h and then incubated with aldosterone, as described for the ALD group. (a) Cells subjected to immunofluorescent assays using DAPI (blue) and FITC (green). (b) Cardiomyocyte surface area (relative to that in the control group) was determined by *α*-actinin immunofluorescence staining. (c) OD values of cardiac fibroblasts were detected by a CCK-8 assay. (d) Hydroxyproline content in the culture supernatant of cardiac fibroblasts. ^△^*P* < 0.05 vs. the blank group; and ^▲^*P* < 0.05 vs. the ALD group.

As shown in Figure 3, the cardiomyocyte surface area, OD value, and hydroxyproline content of the supernatant of cardiac fibroblasts in the ZSHLF group were significantly lower than those in the aldosterone group (*P* < 0.05), suggesting that ZSHLF effectively attenuated the aldosterone-induced hypertrophy of rat cardiomyocytes and inhibited the proliferation and collagen synthesis of cardiac fibroblasts.

### 3.4. Effect of ZSHLF on Striatin mRNA Expression and Interaction between Striatin and the MR Protein in Cardiomyocytes and Cardiac Fibroblasts

**Figure 4:**
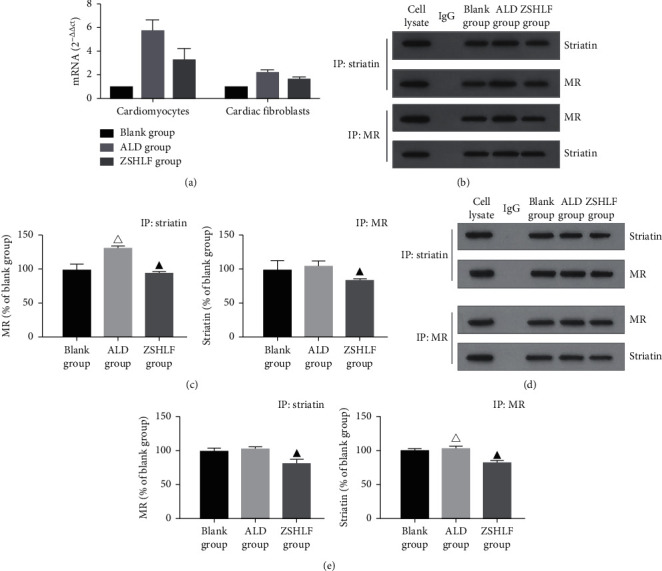
Effect of ZSHLF on striatin mRNA expression and interaction between the striatin and MR proteins in cardiomyocytes and cardiac fibroblasts. In the blank group, the cells were incubated with blank serum-containing medium. In the ALD group, cardiomyocytes and cardiac fibroblasts were pretreated with blank serum, the cardiomyocytes were then incubated with 10^−9^ mol/l aldosterone for 30 min, and the cardiac fibroblasts were incubated with 10^−7^ mol/l aldosterone for 30 min. In the ZSHLF group, cardiomyocytes and cardiac fibroblasts were pretreated with 10% ZSHLF-containing serum for 2 h and then incubated with aldosterone as described for the ALD group. (a) mRNA expression levels of striatin in cardiomyocytes and cardiac fibroblasts were detected by qRT-PCR. The 2^−ΔΔct^ method was used for relative quantitative analysis of mRNA expression levels. (b) Protein bands containing striatin and MR from cardiomyocytes were detected by IP. (c) Protein bands containing striatin and MR from the cardiac fibroblasts were detected by IP. (d) Protein expression of MR and striatin in the cardiomyocytes. (e) Protein expression of MR and striatin in cardiac fibroblasts. ^△^*P* < 0.05 vs. the blank group. ^▲^*P* < 0.05 vs. the ALD group.

As shown in Figure 4(a), qRT-PCR was used to determine the mRNA expression level of striatin in cardiomyocytes and cardiac fibroblasts. In both cell types, the striatin mRNA expression level in the aldosterone group was significantly higher than that in the blank group (*P* < 0.05), while that in the ZSHLF group was lower than that in the aldosterone group (*P* < 0.05).

To detect the interaction between striatin and MR, IP experiments were carried out with antibodies against striatin and MR. As shown in Figures 4(b)–4(e), the MR protein was coprecipitated with antistriatin antibody in the cardiomyocytes and cardiac fibroblasts, and the expression of coprecipitated MR in both cell types pretreated with ZSHLF was significantly lower than that in cells treated with aldosterone alone (*P* < 0.05). In addition, anti-MR antibody coprecipitated with striatin and the expression level of striatin in both cell types pretreated with ZSHLF was lower than that in cells treated with aldosterone alone (*P* < 0.05).

### 3.5. Functional identification of the Role of Striatin in the Nongenomic Effects of Aldosterone on Cardiomyocytes and Cardiac Fibroblasts by siRNA-Mediated Knockdown

**Figure 5:**
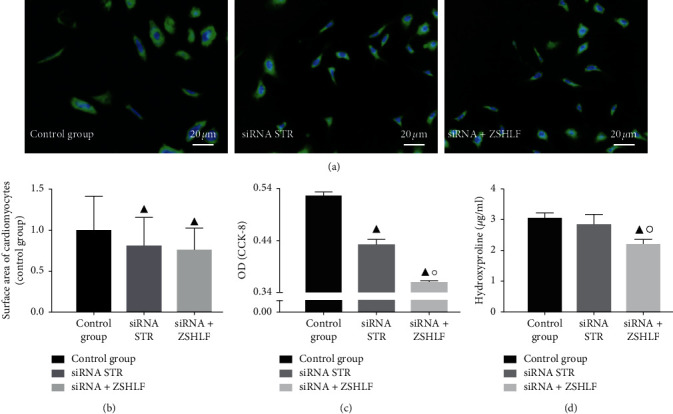
Functional identification of the role of striatin in the nongenomic effects of aldosterone on cardiomyocytes and cardiac fibroblasts by siRNA. In the control group, cardiomyocytes and cardiac fibroblasts were pretreated with blank serum, the cardiomyocytes were then incubated with 10^−9^ mol/l aldosterone for 30 min, and the cardiac fibroblasts were incubated with 10^−7^ mol/l aldosterone for 30 min. Cells in the siRNA STR group were transfected with siRNA STR, pretreated with blank serum for 2 h, and incubated with aldosterone. Cells in the siRNA + ZSHLF group were transfected with siRNA STR, pretreated with ZSHLF-containing serum for 2 h, and then incubated with aldosterone. (a) Cells subjected to immunofluorescent assays using DAPI (blue) and FITC (green). (b) Cardiomyocyte surface area (relative to the control group) determined by *α*-actinin immunofluorescence staining. (c) OD values of cardiac fibroblasts detected by the CCK-8 assay. (d) Hydroxyproline content in the cardiac fibroblast culture supernatant. ^▲^*P* < 0.05 vs. the control group; ^○^*P* < 0.05 vs. the siRNA STR group.

To further verify the role of striatin in the nongenomic effects of aldosterone, we designed and screened three siRNAs against striatin (siRNA STRs), as described in Supplementary Figure 1 and transfected cardiomyocytes and cardiac fibroblasts with the most effective siRNA. The cardiomyocyte surface area in the siRNA STR group was significantly lower than that in the control group (*P* < 0.05), although the cardiomyocyte surface area did not significantly differ between the siRNA STR group and the siRNA + ZSHLF group (*P* > 0.05) (Figures 5(a) and 5(b)). Among cardiac fibroblasts, the OD value in the siRNA STR group was lower than that in the control group, while the OD value in the siRNA + ZSHLF group was lower than that in the siRNA STR group (*P* < 0.05) (Figure 5(c)). The hydroxyproline content in the siRNA + ZSHLF group was not significantly different from that in the control group (*P* > 0.05), while the hydroxyproline content in the siRNA + ZSHLF group was lower than that in the siRNA STR group (*P* < 0.05) (Figure 5(d)).

### 3.6. Effect of ZSHLF on Membrane-Localized MR Protein Expression Levels in Cardiomyocytes and Cardiac Fibroblasts

**Figure 6:**
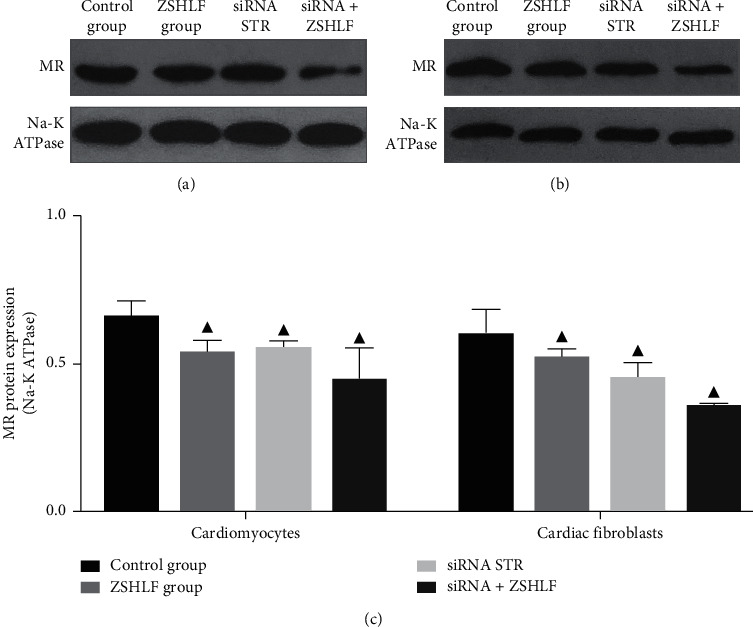
Effect of ZSHLF on membrane-localized MR protein expression levels in cardiomyocytes and cardiac fibroblasts. MR expression levels in cardiomyocytes (a) and cardiac fibroblasts (b) were detected by Western blotting, and the ratio relative to Na-K ATPase expression was used as the relative target protein content (c). ^▲^*P* < 0.05 vs. the control group; ^○^*P* < 0.05 vs. the siRNA STR group.

After the extraction of membrane proteins from cardiomyocytes and cardiac fibroblasts, Western blotting was used to study the role of striatin in regulating the MR protein expression level and the regulatory mechanism of ZSHLF (Figure 6). In both cell types, the MR expression level was lower in the ZSHLF group and the siRNA STR group than in the control group (*P* < 0.05), but the MR expression was not significantly different between the siRNA STR group and the siRNA + ZSHLF group (*P* > 0.05).

### 3.7. Effect of ZSHLF on EGFR, ERK, pEGFR, and pERK Protein Expression in Cardiomyocytes and Cardiac Fibroblasts

**Figure 7:**
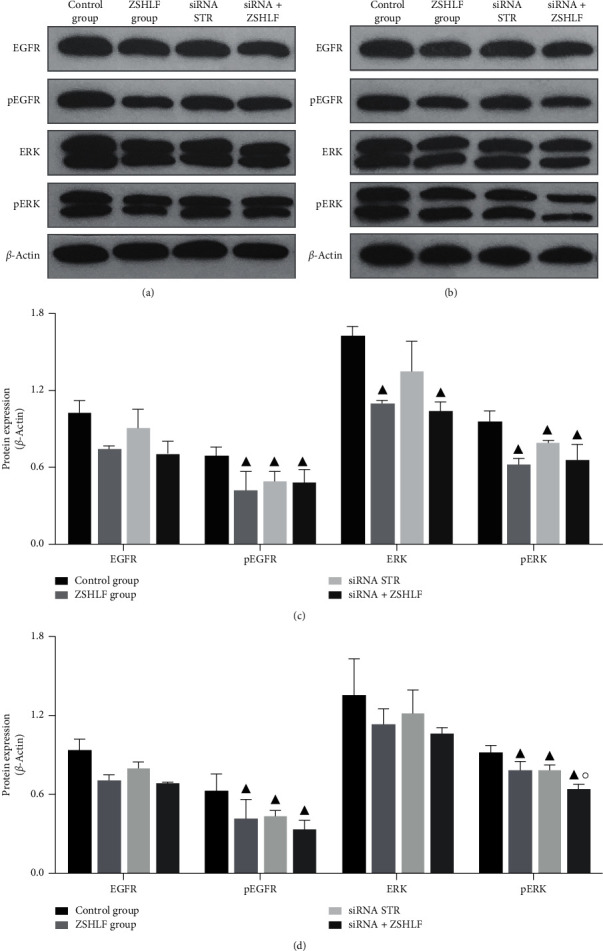
Effects of ZSHLF on the protein expression levels of EGFR, ERK, pEGFR, and pERK in cardiomyocytes and cardiac fibroblasts. Expression levels of EGFR, ERK, and phosphorylated proteins in cardiomyocytes (a) and cardiac fibroblasts (b) were detected by Western blotting, and the ratio relative to *β*-actin expression was used as the relative target protein content (c). ^▲^*P* < 0.05 vs. the control group; ^○^*P* < 0.05 vs. the siRNA STR group (d).

The Western blot analysis of cardiomyocytes did not identify significant differences in EGFR protein expression among the groups (*P* > 0.05). The pEGFR and pERK expression levels were lower in the ZSHLF group and siRNA STR group than the control group, the ERK expression levels were significantly lower in the ZSHLF group than the control group (*P* < 0.05), and the pEGFR, ERK, or pERK expression levels did not significantly differ between the siRNA STR group and siRNA + ZSHLF group (*P* > 0.05) (Figures 7(a) and 7(c)).

The Western blot analysis of cardiac fibroblasts revealed that the EGFR and ERK expression levels did not significantly differ among the groups (*P* > 0.05). The pEGFR and pERK expression levels were lower in the ZSHLF group and siRNA STR group than in the control group (*P* < 0.05), the pERK expression level was lower in the siRNA + ZSHLF group than the siRNA STR group (*P* < 0.05), and the pEGFR expression did not obviously differ between the siRNA + ZSHLF group and the siRNA STR group (*P* > 0.05) (Figures 7(b) and 7(d)).

## 4. Discussion

Long-standing hypertension leads to LVH, a recognized risk factor for heart failure, myocardial infarction, arrhythmia, sudden cardiac death, and stroke [18, 19]. The reversal of LVH as antihypertensive therapy can improve the prognosis and reduce the morbidity and mortality of cardiovascular disease [20]. ZSHLF is an empirical prescription for the clinical treatment of hypertension. Animal and clinical studies have shown that the combination of ZSHLF and perindopril can steadily reduce blood pressure and inhibit the pathological changes in myocardial hypertrophy and myocardial fibrosis, which effectively control the LVH process [15]. In this study, ZSHLF significantly inhibited aldosterone-induced cardiomyocyte hypertrophy and proliferation and collagen synthesis in cardiac fibroblasts, and the associated mechanism may be closely related to the nongenomic effect of aldosterone, which affects the downstream EGFR/ERK signaling pathway by regulating the interaction between striatin and MR. We identified five main chemical components of ZSHLF-containing serum as betaine, caffeic acid, DL-stachydrine, catechin, and coumarin; however, the components associated with the improvement of aldosterone-induced myocardial remodeling need to be further identified.

The RAAS is an important link between the regulation of blood pressure and electrolyte balance in the human body [21]. A sustained increase in aldosterone after chronic activation of the RAAS can lead to hypertension, thrombosis, and atherosclerosis and promote inflammation [22, 23]. In the classical pathway, aldosterone binds cytoplasmic MR, which is transferred to the nucleus, where it dimerizes and exerts genomic effects as a transcription factor. In recent years, a great deal of evidence has demonstrated that aldosterone also triggers a very rapid response in the typical epithelial target organs of MR and nonepithelial tissues [17, 24]. As this reaction is often completed in a few minutes and does not require transcription or translation, it is called a nongenomic effect [25]. In this study, cardiomyocytes and cardiac fibroblasts were treated with aldosterone. Aldosterone induced cardiomyocyte hypertrophy, fibroblast proliferation, and hydroxyproline secretion, and these effects were initially observed at 5 min and peaked at 30 min. The short-term nature of these effects is consistent with the characteristics of a nongenomic action. However, in all three groups, the effect of aldosterone induction at 2 h was stronger than that at 1 h, suggesting a simultaneous genomic effect. The main purpose of this study was to explore the nongenomic effect of ZSHLF; therefore, an induction time of 30 min was chosen to further study the regulatory effect of ZSHLF. ZSHLF had an obvious inhibitory effect on the aldosterone-induced increase in cardiomyocyte surface area and the proliferation and hydroxyproline secretion of cardiac fibroblasts, suggesting that ZSHLF can improve myocardial remodeling by regulating the nongenomic effect of aldosterone.

The striatin protein, which is widespread in the heart, kidney, and blood vessels, is a multidomain scaffold protein that can interact with and activate different signal transduction molecules [26], participate in membrane-related signal transduction, and regulate the rapid nongenomic and genomic effects of steroids. Striatin is an important intermediary of the nongenomic effect of aldosterone [27], and evidence suggests that the cytosolic MR protein is partially transported near the cell membrane after it binds striatin [28, 29]. Formation of a complex between striatin and MR in endothelial cells and mouse heart tissues was shown to be related to the nongenomic effect of aldosterone [30, 31]. We speculated that ZSHLF inhibits aldosterone-induced myocardial remodeling by regulating striatin gene expression and interacting with MR. The qRT-PCR assay showed that in both cardiomyocytes and cardiac fibroblasts, striatin mRNA expression was increased in response to aldosterone induction; however, ZSHLF pretreatment restrained this change to some extent. The IP assay confirmed that striatin and MR interacted in rat cardiomyocytes and cardiac fibroblasts and MR/striatin complex formation was weakened by ZSHLF pretreatment. To further verify the role of striatin in the nongenomic effects of aldosterone on cardiomyocytes and cardiac fibroblasts, we used siRNA to reduce striatin gene expression. Striatin silencing decreased the cardiomyocyte surface area and the proliferation rate of cardiac fibroblasts but had no significant effect on the hydroxyproline content. The suppressive effect of ZSHLF treatment combined with siRNA transfection on the cardiomyocyte surface area was not significantly different from that of siRNA transfection alone, whereas the effect on the proliferation rate and hydroxyproline content of cardiac fibroblasts was more pronounced under the combination of ZSHLF treatment and siRNA transfection than with siRNA transfection alone. These observations suggest that aldosterone induces cardiomyocyte hypertrophy and cardiac fibroblast proliferation through the striatin/MR complex, and that ZSHLF inhibits cardiomyocyte hypertrophy and cardiac fibroblast proliferation by reducing the binding of striatin and MR. Furthermore, ZSHLF may also regulate cardiac fibroblast proliferation and collagen synthesis through targets independent of striatin.

**Figure 8:**
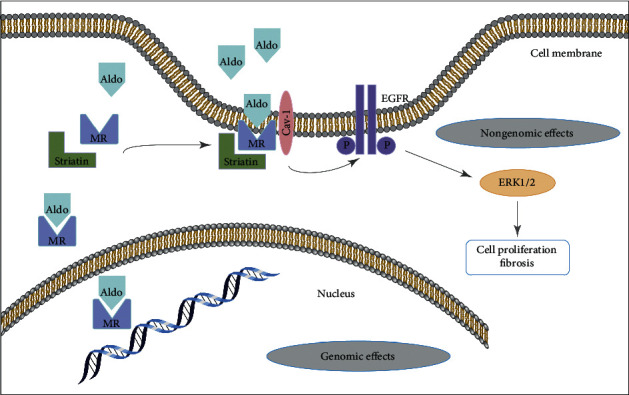
Hypothetical model describing the striatin-mediated signaling pathway. After binding striatin, MR is translocated to and associated with the cell membrane to form a signaling complex. On aldosterone stimulation, the EGFR/ERK signaling pathway is activated, resulting in cell proliferation and fibrosis.

EGFR is a member of the receptor tyrosine kinase family and plays a key role in regulating cell proliferation, differentiation, and survival. Its interaction with aldosterone/MR has been identified as a potential cause of its nongenomic effect, which likely proceeds through a molecular mechanism that mediates the physiological and pathological effects of aldosterone [32]. MR is transported near the cell membrane by striatin, where it forms a signaling complex with caveolin, src, and EGFR [28, 29], which induces the crosslinking of ERK1/2 and the phosphoinositide-3-kinase/mechanistic target of rapamycin by activating EGFR signaling and induces fibroblast proliferation, thereby further aggravating fibrosis [13, 33]. In addition, in human and mouse endothelial cells, the rapid effects of MR on ERK phosphorylation require striatin, and a decrease in striatin levels leads to a reduction in aldosterone/MR-dependent ERK phosphorylation [17]. Based on the above studies, we examined the role of the MR/EGFR/ERK signaling pathway in the striatin-mediated nongenomic effects of aldosterone and the regulatory effect of ZSHLF in cardiomyocytes and cardiac fibroblasts. After striatin silencing, the protein expression levels of membrane-localized MR and the levels of phosphorylated EGFR and ERK1/2 in cardiomyocytes and cardiac fibroblasts decreased. The ZSHLF treatment of striatin-silenced cells did not significantly alter the protein expression level of MR, the level of EGFR phosphorylation in cardiomyocytes or cardiac fibroblasts, or the levels of ERK1/2 phosphorylation in cardiomyocytes, whereas it did reduce ERK1/2 phosphorylation in cardiac fibroblasts. Therefore, we speculate that striatin induces the phosphorylation of EGFR and ERK by mediating the membrane localization of MR, which results in aldosterone-induced cardiomyocyte hypertrophy and cardiac fibroblast proliferation (Figure 8). ZSHLF inhibits cardiomyocyte hypertrophy and cardiac fibroblast proliferation through the MR/EGFR/ERK signaling pathway mediated by striatin. In addition, ZSHLF may inhibit the proliferation and collagen synthesis of cardiac fibroblasts by regulating the ERK signaling pathway through other targets independent of striatin.

## 5. Conclusion

In summary, ZSHLF-containing serum effectively prevented aldosterone-induced cardiomyocyte hypertrophy, cardiac fibroblast proliferation, and collagen synthesis; inhibited the downstream EGFR/ERK signaling pathway by blocking striatin-mediated localization of MR to the membrane; and altered the nongenomic effects of aldosterone, and these effects may all represent important molecular mechanisms by which ZSHLF improves hypertensive myocardial remodeling. Notably, ZSHLF also inhibited the proliferation and collagen synthesis of cardiac fibroblasts by regulating other targets independent of striatin. These additional targets and signaling mechanisms are worthy of further study and discussion. The identification of the main components of ZSHLF associated with the improvement of aldosterone-induced myocardial remodeling is also one of the directions of our next work.

## Figures and Tables

**Table 1:** Primer sequences for qRT-PCR assay.

Primer name	Primer sequence (5′–3′)	Product length
Striatin-F	GAGACAACGAGTCCAGAAG	222
Striatin-R	GCAAGGAGGGAAGTTCATC	
GADPH-F	GTCGGTGTGAACGGATTTG	181
GADPH-R	TCCCATTCTCAGCCTTGAC	

## Data Availability

The data used to support the findings of this study are available from the corresponding author upon request.

## Abbreviations

ZSHLF: Zi Shen Huo Luo Formula
MR: Mineralocorticoid receptor
GFR: Epidermal growth factor receptor
ERK: Extracellular signal-regulated kinase
LVH: Left ventricular hypertrophy
siRNA: Small interfering RNA
RAAS: Renin-angiotensin-aldosterone system
ACEI: Angiotensin converting enzyme inhibitor
qRT-PCR: Quantitative real-time PCR
IP: Immunoprecipitation
UPLC: Ultraperformance liquid chromatography
MS: Mass spectrometer.

## Supplementary Materials

Supplementary Materials To verify the role of striatin in the nongenomic effects of aldosterone, we designed and screened three siRNAs against striatin. Because the purpose of this experiment is to screen the most effective siRNA, which could be used in subsequent experiments to knockdown striatin expression. Supplemental Figure 1: identification and screening of striatin siRNA interference efficiency.
