# Supplementary material for: Zi Shen Huo Luo Formula Prevents Aldosterone-Induced Cardiomyocyte Hypertrophy and Cardiac Fibroblast Proliferation by Regulating the Striatin-Mediated MR/EGFR/ERK Signaling Pathway
Source: Evid Based Complement Alternat Med. 2020 Sep 16;2020:9028047. doi: 10.1155/2020/9028047 (PMC7519188; doi:10.1155/2020/9028047)
Supplement: Supplementary Materials — To verify the role of striatin in the nongenomic effects of aldosterone, we designed and screened three siRNAs against striatin. Because the purpose of this experiment is to screen the most effective siRNA, which could be used in subsequent experiments to knockdown striatin expression. Supplemental Figure 1: identification and screening of striatin siRNA interference efficiency. [file 9028047.f1.docx]

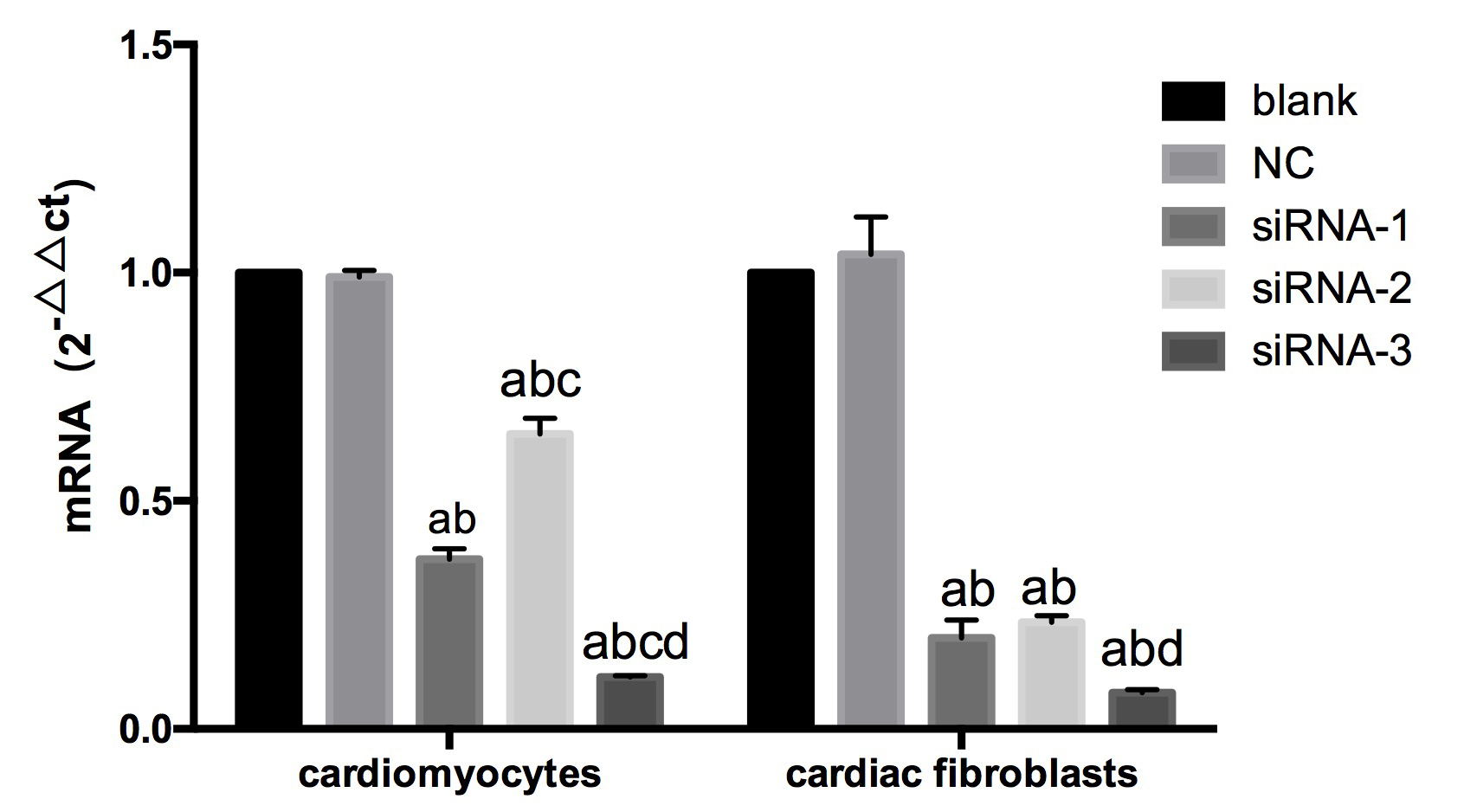


FIGURE 1: *Identification and screening of striatin siRNA interference efficiency.* Cardiomyocytes and cardiac fibroblasts were transfected with three siRNAs against striatin for 24 h, and the most efficient siRNA was identified by qRT-PCR. The relative copy numbers in cardiomyocytes and cardiac fibroblasts transfected with siRNA-1, siRNA-2 and siRNA-3 were significantly lower than those in the blank group and NC group, and those in the siRNA-3 group were significantly lower than those in the siRNA-1 and siRNA-2 groups (*P*<0.05). There was no significant difference in copy number between the blank group and NC group. These data suggest that the siRNA design was reasonable and that the siRNAs effectively inhibited striatin gene expression in cardiomyocytes and cardiac fibroblasts. The siRNA-3 interference efficiency was the greatest; therefore, siRNA-3 was used for follow-up studies. ^a^*P*<0.05, vs. the blank group; ^b^*P*<0.05, vs. the NC group; ^c^*P*<0.05, vs. the siRNA-1 group; ^d^*P*<0.05, vs. the siRNA-2 group.
